# FDA Approved Drugs Repurposing of Toll-Like Receptor4 (TLR4) Candidate for Neuropathy

**DOI:** 10.22037/ijpr.2019.2394

**Published:** 2019

**Authors:** Hakimeh Zali, Ali Golchin, Masoumeh Farahani, Mohsen Yazdani, Mohammad Mehdi Ranjbar, Ali Dabbagh

**Affiliations:** a *Department of Tissue Engineering and Applied Cell, School of Advanced Technologies in Medicine, Shahid Beheshti University of Medical Sciences, Tehran, Iran.*; b *Proteomics Research Center, Faculty of Paramedical Science, Shahid Beheshti University of Medical Sciences, Tehran, Iran.*; c *Institute of Biochemistry and Biophysics, Tehran University, Tehran, Iran.*; d *Razi Vaccine and Serum Research Institute, Agricultural Research, Education and Extension Organization, Karaj, Iran.*; e *Anesthesiology Research Center, Shahid Beheshti University of Medical Sciences,Tehran, Iran.*

**Keywords:** Approved drugs, Docking, Pathway studio, TLR4, Peuropathic pain, Virtual-screening

## Abstract

Accumulating evidence indicates that toll-like receptor 4 (TLR4) plays a critical role in promoting adaptive immune responses and are definitively involved in the expansion and maintenance of the neuropathic pain. Though the application of docking in virtual-screening *in silico* methods to drug discovery has some challenge, it allows directed and meaningful design of drugs for a target protein; which can lead to low costing approaches with shortcuts; resulting in evolution and discovery of promising new drugs. Nevertheless, in parallel with virtual screening methods, attendant developments in cell culture and *in-vivo* studies must be achieved. In the present paper, we aimed to discover new drugs that have the ability to bind and inhibit TLR4 functions. So, after using the Pathway studio to investigate the biological pathways and protein interaction maps between TLR4 and neuropathy, we reported the application of the affinity-based approach of different pharmaceuticals; these agents contained all of the approved drugs; which could bind to Toll-like receptor 4 in blind high-throughput *in silico* screening. Our results demonstrated that among the primary list of 1945 retrieved compounds, 39 approved compounds could be the right candidate to perform a biological test in different *in-vivo* and *in-vitro* conditions and as a lead for further neurophysiological and neuropathological studies and treatment of neuropathic pain.

## Introduction

**Figure 1:**
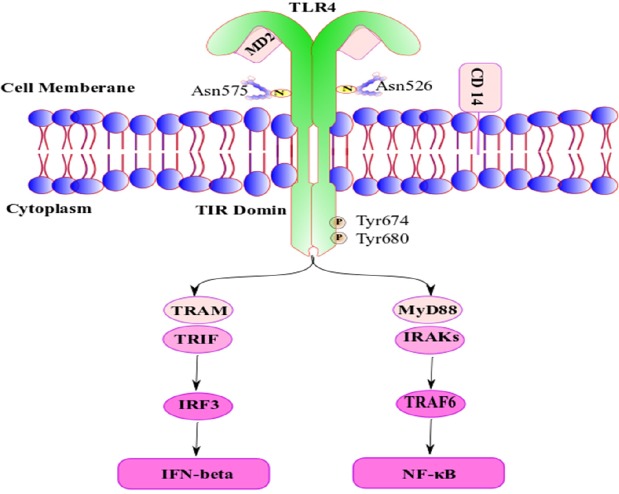
A schematic representation of Structure of TLR4/MD-2 complex and its signaling after activation

Toll like receptors are members of the pattern recognition receptor (PRR) family; with a key role in innate immunity system. Their main role is to detect pathogen molecules and initiate an immunologic response to them (1) especially through lipopolysaccharide recognition, production of pro-inflammatory cytokines and increased levels of type I interferon production (2). Therefore, it can play an important role in various physiological and pathological processes such as septic shock and other inflammatory disorders (3, 4). Toll-Like Receptor 4 (TLR4) is one of the PRR family members, whose cytogenetic location is chr9: q33.1 - q33.1 and its alternate symbols are ARMD10; CD284; TLR-4; hToll. TLR4 in mice can be mediated plasma lipopolysaccharide (LPS) function to induce pro-inflammatory cytokines release and apoptosis in cultured myenteric neurons (Figure 1) (5-7). TLR4 exist to a great extent in the cell surface and endosomes of immune and non-immune cells such as monocytes, marcophages (8-10), dendritic cells (11, 12), mast cells (13), Intestinal epithelium (14), and pancreatic β-cells (15), and also it is widely expressed in human nervous system, including by DRG neurons, astrocytes, and microglia, as well as endothelial cell (3, 4, 16).

*Structure*

TLR4 is a dimeric type I transmembrane receptor. Glycosylated Asn526 and Asn575 is necessary for the expression TLR4 on the cell surface after translation in inside the cell, and phosphorylation of Tyr674 and Tyr680 is necessary for optimal TLR4 signal transduction. 

*Signaling*

CD14 (cluster of differentiation 14) is the receptor of the complex formed by lipopolysaccharide (LPS) with LBP (LPS binding protein) and via attaching to TLR4/DM2 enhancing signal transduction by TLR4. TLR4 activation can signal via TIR-domain-containing adapter-inducing interferon-β (TRIF) dependent pathways via the adaptor TRIF-related adaptor molecule (TRAM).

TRIF activates Interferon Regulatory Factor 3 (IRF3) and initiates the production of IFN (interferon) type I. Alternatively, myeloid differentiation factor 88 (MyD88) interacts with Interleukin-1R-Associated Kinases (IRAK′s) to activate TNF-receptor-associated factor-6 (TRAF6); the next step is activation of nuclear factor κB (NF-κB); however, the final step of these activated pathway is induction of pro-inflammatory mediators (16). 

*TLR4 Signaling in Nervous System*

TLR4 act in complexes with CD14 and MD-2, and it can be activated by several structurally diverse ligands such as LPS, Taxol, Heparan, Hyaluronate, F-prot, RSV, G-prot, VSV, Env prot, and etc. (8). In addition, Four adapters (MyD88, Tirap TRIF,TRAM) are known to serve the Toll/Interleukin-1 receptor (TIR) domain receptors for TLR4 signaling (8, 17). MD-2 is a small-secreted protein that is stoutly associated with the TLR4 ectodomain to create the TLR4–MD2–LPS complex, so it is essential in TLR4 activation (17).

TLR4 signaling is different pathways in different neural cell types (18, 19), For example, in astrocytes, TLR4 activates the MyD88-dependent pathway, and MyD88-mediated signaling leads to the transcription of TNF-α, vascular cell adhesion molecule 1 (VCAM-1) and IL-27, even so, other signaling mediators activation transcribe IP-10, suppressor of cytokine signaling proteins-1 (SOCS-1), IL15, and matrix metalloproteinase MMP9 (20, 21). In other neuronal system cell types like microglia, neural progenitor cells (NPCs), and dendritic cells, TLR4 activation included MyD88- and TRIF-dependent signaling pathways, although there were differences in each one; see figure in the review by Okun *et al*. (20). Recent evidence indicates that peripheral nerve injury can induce spinal microglia/astrocytic activation with a pivotal role of toll-like receptor 4 (TLR4) in several chronic neuropathic pain models (22); so that TLR4-knockout and point mutant mice developed less neuropathic pain and strongly decreased expression of pain related cytokines (23). LPS and endogenous ligands for TLR4, lead to NF-κB activation and subsequent induction of pro-inflammatory cytokines, which contribute to neuropathy pain process (23, 24). So, Microglial TLR4 plays a crucial part as a receptor in the induction phase of behavioral hypersensitivity in rodent models of neuropathy (18, 22, 23, 25). However, the signaling and role of neuronal TLR4 in innate neuro-immunity and painful neuropathy remains completely unknown.

As mentioned above, Toll-Like Receptor 4 (TLR4) is one of the prominent members of Toll-Like Receptors, having a critical role as a mediator of neuropathic pain throughout the process of peripheral nerve injury. Pharmacological TLR4 inhibition provides partial protection against neuropathic pain; while inhibition of TLR4 could be a promise in controlling and treatment of many disease states leading to novel drug discovery (1, 9, 26-30). Therefore, in the current study, we report the application of the affinity-based approach of different pharmaceuticals (from the drug bank: www.drugbank.ca, as of July 2017, and contains all of the approved drugs.) binding to Toll-like receptor4 in blind high-throughput *in silico* screening for the discovery of new drugs that have the ability to bind and inhibit TLR4 functions.

## Experimental

*Pathway Studio*

We run the neuropathy and TLR4 keywords in the pathway studio database (https://www.pathwaystudio.com/) to obtain a combined pathway from the network enrichment analysis of pathway studio and evaluate the interaction between TLR4 and neuropathy conditions. The aim of this stage of our study was an investigation of the recent different literature reviews to realize the different interaction between TLR4 and neuropathy and to find used compounds, which may have antagonist effect for TLR4 and can inhibit neuropathy.

**Figure 2:**
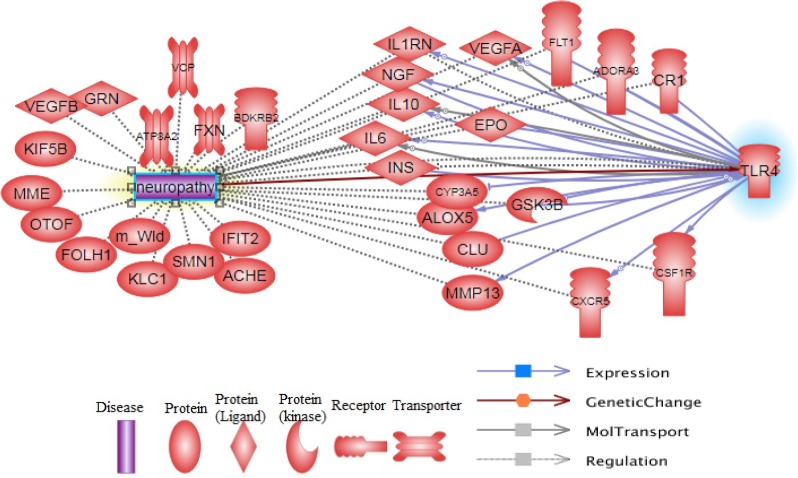
Pathway constructed from the Pathway studio database and show main functional proteins that linked between TLR4 and neuropathy

**Figure 3:**
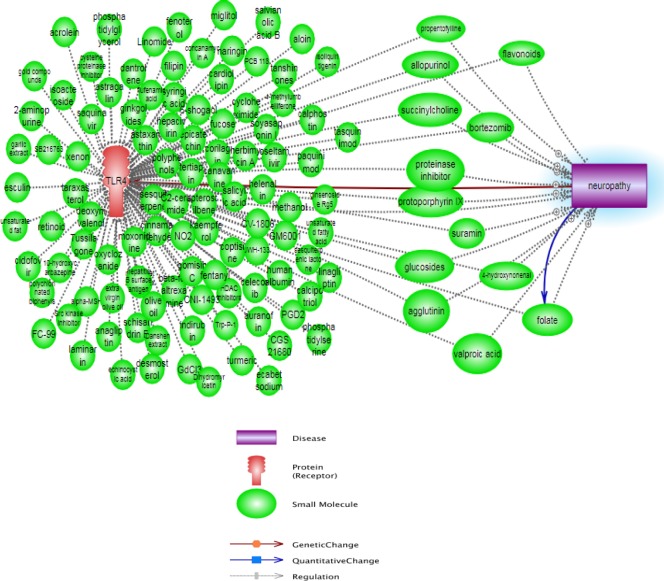
Pathway constructed from the Pathway studio database and show affected small molecules between TLR4 and neuropathy pathways

**Table 1:** Observed binding affinities of the approved drugs to the TLR4 inhibition

**Drug Name**	**DrugBank ID**	**Affinity**
Dutasteride	DB01126	-12.3
Buclizine	DB00354	-11.6
Conivaptan	DB00872	-11.5
Lomitapide	DB08827	-11.4
Adapalene	DB00210	-11.2
Lumacaftor	DB09280	-11.2
Dihydroergotamine	DB00320	-11
Bromocriptine	DB01200	-11
Tolvaptan	DB06212	-11
Nandrolone phenpropionate	DB00984	-10.9
Flunarizine	DB04841	-10.9
Irinotecan	DB00762	-10.8
Dihydrotachysterol	DB01070	-10.8
Antrafenine	DB01419	-10.7
Nilotinib	DB04868	-10.7
Medrogestone	DB09124	-10.6
Itraconazole	DB01167	-10.4
Doxercalciferol	DB06410	-10.4
Ponatinib	DB08901	-10.4
Netupitant	DB09048	-10.4
Difenoxin	DB01501	-10.3
Paricalcitol	DB00910	-10.2
Pimozide	DB01100	-10.2
Tasosartan	DB01349	-10.2
Alfacalcidol	DB01436	-10.2
Fluspirilene	DB04842	-10.2
Azilsartan medoxomil	DB08822	-10.2
Cabozantinib	DB08875	-10.2
Lorpiprazole	DB09195	-10.2
Ergocalciferol	DB00153	-10.1
Loperamide	DB00836	-10.1
Drospirenone	DB01395	-10.1
Pranlukast	DB01411	-10.1
Ziprasidone	DB00246	-10
Meclizine	DB00737	-10
Sertindole	DB06144	-10
Lurasidone	DB08815	-10
Vorapaxar	DB09030	-10
Umeclidinium	DB09076	-10

*Molecular structures and energy / Minimization/ Protein preparation*

The X-ray crystal structure of toll-Like Receptor 4 in complex MD2 (TLR4/MD2) (PDB ID 3FXI) was retrieved from protein data bank (www.pdb.org). Then, the water molecules were removed and the receptor cleaned from any unwanted interactions. The selected structure was energetically minimized by SPDV viewer software tool with GROMOS96 implementation. For correct ionization and tautomeric states of amino acid residues, all nonpolar hydrogens were merged (removed) and partial atomic charges were assigned using the Gasteiger-Marsili method. Then, the charges were added to receptor structures and Kollman United Atom charges and atomic salvation parameters were assigned. The binding site and surface of receptor were detected based on the previous reported data.

*Ligand preparation*

1948 compounds were retrieved from the DrugBank database (www.drugbank.ca, as of July 2017) from the small-molecules section, containing all the approved drugs. The data were curated using the following protocol:

1. The inorganic compounds (not containing carbon atoms) were removed

2. The mixtures were separated and salts were removed

3. The organometallic compounds were removed

4. The compounds containing rare atoms (selenium, silicon, gold, platinum) were removed

5. The non-unique structures were removed; and 

6. The compounds permanently charged were removed

The software used for these different tasks was ChemAxon’s Instant JChem v.5.3 (http://www.chemaxon.com).

After curation, the screening database contained 1553 compounds. Briefly, the Hyperchem software (ver. 7.0) was used to generate 3D molecular structures and minimize their energy using MM+ force field (27). Then, the structures were fully optimized based on the semi-empirical method, using AM1 level of theory

*Molecular docking*

Molecular docking was carried out in order to evaluate a possible binding mode between drugs and TLR4 binding sites. Docking in virtual-screening gives suitable indication of the possible biological activities of the compounds and reduces cost and time of drug discovery. Moreover, it estimates the strength of the binding, the energy of the complex and also calculates the binding affinity using scoring functions. The docking studies were performed using AutoDock Vina (31). This software is an academic-free molecular docking and virtual screening software, which works based on empirical scoring functions. AutoDock Vina can compute the grid maps in an automatic manner (31, 32).

This study was financially supported by Shahid Beheshti University of Medical Sciences, Tehran, Iran (the research project coded 9237). The study was ethically approved by Research Ethics Committee, Deputy of Research, Shahid Beheshti University of Medical Sciences, Tehran, Iran. The ethics code was IR.SBMU.REC.1396.16.

## Results

The pathway of regulation and expression of the main involved proteins in neuropathy and TLR4 function is represented in Figure 2.

Additionally, the affected compounds and small molecules between TLR4 actions and neuropathy pathway are shown in Figure 3, which none of them did not have antagonism effect in TLR4 and neuropathy mechanisms. As seen, the emerging advances in computer-aided drug design allow us to make the directed and meaningful design of drugs for a target protein; this is a shortcut and low cost method for evolution and discovery of many promising new drugs. We estimated the binding affinity using scoring functions and Table 1 shows the drugs with TLR4 antagonist activities which are the result of this study.

## Discussion

The TLR4 is a member of the Toll-like receptor (TLR) family which plays a fundamental role in pathogen recognition and activation of innate immunity. furthermore, the MD-2 is a glycoprotein that is essential for the innate response to lipopolysaccharide (LPS), binds to both LPS and the extracellular domain of Toll-like receptor 4 (TLR4) (33). 

Despite all the investigation, only one approved drug exists in Drug bank site (https://www.drugbank.ca/biodb/bio_entities/BE0002442). Naloxone has lacked opioid receptor affinity and selective for TLR4 inhibition, so each of the TLR4 antagonists (+)-naloxone and (-)-naloxone can fully reverse the established neuropathic pain upon multi-day administration (34).

*Inhibition of TLR4 and in silico studies*

The most used docking tools for virtual screening in the TLRs field have included Glide, AutoDock VINA, GOLD, Surflex-dock, FlexX, ICM, and DOCK. In one study, Gobec et al, used the ZINC drug-like subset (~11.3 million drug-like compounds) from the ZINC database for ligand-based virtual screening by using the OMEGA software, finally three compounds identified as TLR4 inhibitors (ZINC: ZINC49563556, ZINC: ZINC3415865, ZINC: ZINC25778142) Also, five not active compounds were identified: ZINC51408124, ZINC464832, ZINC26905159, ZINC32525142, and ZINC32524933 which were evaluated to *in-vitro* study on HEK-BlueTM-hTLR4 reporter cell line, and unfortunately, the results of *in-vitro* study were not suitable and applicable (35). In another study, about 86,000 clusters were isolated from ENAMINE database, and investigated by a novel *in silico* screening methodology incorporating Molecular Mechanics (MM)/implicit solvent methods to evaluate binding free energies. After the compounds were screened against both TLR4 and MD-2, two compounds, T5342126 and T6071187 were identified as potential TLR4- and MD-2-specific inhibitors, which were completely abolishing LPS-induced activation of signaling (36). In the other virtual screening and *in silico* studies that were done, respectively, these compound were identified as TLR4 modulators (36): ENAMINE: T5342126, ENAMINE: T6071187 (37), ZINC: ZINC04272679, ZINC: ZINC00611718, ZINC: ZINC04272561, ZINC: ZINC48141941, ZINC: ZINC09535665, ZINC: ZINC70039563, ZINC: ZINC29450369, ZINC: ZINC64951618, ZINC: ZINC41124663, ZINC: ZINC08687988, ZINC: ZINC64951738, ZINC: ZINC72278680 (37), C34 (38). 

However, some inhibitor compounds and drugs have been introduced in different studies: Eritoran, TAK-242, arylidene malonate derivatives, Tanshinone II, Naltrexone, and Ligustilide (39-43).

In our study, among different pharmaceuticals libraries, with regard to molecular docking results, the 39 potent TLR4 inhibitors have been identified and proposed. Our results demonstrated that these drugs could be a good candidate to perform biological test in different *in-vivo* and *in-vitro* conditions and as a lead for further developments. The results presented in the current study indicate that the application of *in silico* study guarantees the best predictive methods, so our results can shorten the evolution of several very promising lead new drugs because these drugs have passed the direction of the approved drugs from Food and Drug Administration (FDA). Our study paves the way to a broader application of docking in virtual-screening in TLR4 inhibitor drug discovery.
